# An Open Personal Letter to the Members of the I. H. A.

**Published:** 1891-11

**Authors:** James B. Bell

**Affiliations:** Boston


### AN OPEN PERSONAL LETTER TO THE MEMBERS OF THE I. H. A.

DEAR COLLEAGUES:

I think I ought not to wait until the next meeting to express to you my thanks for the honor of being chosen President of the Association in my absence, and without any desire or knowledge on my part.

Indeed, I must frankly say, that much as I esteem the honor and prize most highly the recognition of my co-workers which is thus expressed, I still think the Association was more kind than wise.

I have very little aptitude and less taste for executive or parliamentary business. I had besides several things in hand for the Association, which I had been unable to complete thus far, though I hoped to do so this year.

One of these things is some surgical work. Another, on which I have quite set my heart, something of the nature of "Hints to Patients" for the use of all the members, in convenient form and size for distribution; containing as much instruction, warning, and advice on our principles and practice as can be wisely made public; put in as pithy and telling a style as possible, for the making of more obedient, intelligent, and faithful patients.

If some one else will take this work up now I will be very glad. I think, with all our best efforts, we are not making the headway we ought, as far as creating a permanent constituency is concerned. Too many patients are content with the man rather than with his practice, and often change schools if they change their residence. I know that all of us are doing much orally in this way; but it takes lots of talk and precious time, and then the subject is not finished with any one patient.

When is the time to begin work for the Bureau? Eminently *now*.

Now is always a good time, but especially because work laid out now and finished as time permits will be better done than if left until the warm days and short evenings of May or June.

You will doubtless hear from our able and enthusiastic Chairman shortly, but let us not wait for that. Remember that therapeutically we are the salt of the earth as well as the light thereof.

And we can salt quite a little amount of territory now if we exert ourselves, and light up a great deal of darkness, if we let our *light shine*.

Yours fraternally,

JAMES B. BELL.

BOSTON, Oct. 1st, 1891.

